# Mitochondrial dysfunction of induced pluripotent stem cells-based neurodegenerative disease modeling and therapeutic strategy

**DOI:** 10.3389/fcell.2022.1030390

**Published:** 2022-11-21

**Authors:** Hong-Mei Luo, Jia Xu, Dan-Xia Huang, Yun-Qiang Chen, Yi-Zhou Liu, Ya-Jie Li, Hong Chen

**Affiliations:** ^1^ Department of Rehabilitation, Tongji Hospital, Tongji Medical College, Huazhong University of Science and Technology, Wuhan, China; ^2^ Stem Cell Research Center, Tongji Hospital, Tongji Medical College, Huazhong University of Science and Technology, Wuhan, China

**Keywords:** neurodegenerative diseases, mitochondrial dysfunction, therapeutic strategy, IPSCs (induced pluripotent stem cells), mitochondrial dynamic, mitochondrial transport, mitochondrial energy metabolism

## Abstract

Neurodegenerative diseases (NDDs) are disorders in which neurons are lost owing to various factors, resulting in a series of dysfunctions. Their rising prevalence and irreversibility have brought physical pain to patients and economic pressure to both individuals and society. However, the pathogenesis of NDDs has not yet been fully elucidated, hampering the use of precise medication. Induced pluripotent stem cell (IPSC) modeling provides a new method for drug discovery, and exploring the early pathological mechanisms including mitochondrial dysfunction, which is not only an early but a prominent pathological feature of NDDs. In this review, we summarize the iPSC modeling approach of Alzheimer’s disease, Parkinson’s disease, and Amyotrophic lateral sclerosis, as well as outline typical mitochondrial dysfunction and recapitulate corresponding therapeutic strategies.

## 1 Introduction

With the aging of the population, neurodegenerative diseases (NDDs) are affecting an increasing number of people. By 2030, seventy-eight million individuals would have been affected by dementia (predominantly Alzheimer’s disease), with up to 55 million cases by 2021 (WHO, 2021). NDDs are characterized by the progressive loss of motor, sensory, and/or cognitive functions as a result of neuronal cell death (Pasteuning-Vuhman et al., 2021). Several common NDDs, including Alzheimer’s disease (AD), Parkinson’s disease (PD), and amyotrophic lateral sclerosis (ALS), have a remarkably complex pathogenesis. Due to its high etiologic heterogeneity, interplayed with diverse genetic backgrounds, and environmental factors, there are few target strategies despite decades of research (Giacomelli et al., 2022). Diving into the pathogenesis of NDDs remains a prioritized step toward their understanding and treatment.

Although several models have been developed to aid mechanistic research, much remains elusive (Ke et al., 2021; Okano and Morimoto, 2022). The autopsy sample serves as an ideal model for investigation; however, it is featured as the late stage of NDDs, which is unfit for early pathophysiological studies. Animal models have been utilized in numerous studies, but the translational clinic applications remain a long way off (Okano and Morimoto, 2022). With such a predicament, it is essential to develop accurate models that reflect the early stages of the pathological processes in NDDs (Israel and Goldstein, 2011; Grekhnev et al., 2022).

Induced pluripotent stem cells (iPSCs) are pluripotent, which indicates their capacity to differentiate into almost all human cell types with identical genetic background of the donor patient (Li and Fraenkel, 2021). This process provides developmental cues to areas of interest. Moreover, patient-specific iPSCs combined with gene editing technology can also be used for the development of therapeutic targets and clinical drug screening (Okano and Yamanaka, 2014; Park et al., 2014). NDDs affect specific types of neurons; therefore, the *in vitro* modeling of patient-specific iPSCs to thoroughly understand the pathological processes of NDDs has significantly furthered the exploration of their mechanism.

Mitochondria are organelles that continuously undergo fusion and fission in eukaryotic cells and play a crucial role in bioenergetic and biosynthetic pathways, calcium homeostasis management, and the regulation of programmed cell death (Friedman and Nunnari, 2014). Mitochondrial dysfunction has emerged as one of the major participants in NDDs (Magrané et al., 2012; Shaltouki et al., 2015; Fang et al., 2019). For instance, mitochondrial transport disturbances were found in ALS SOD1^G93A^MN (Magrané et al., 2012). Fang and his team observed the accumulation of damaged mitochondria in the hippocampus of AD patients due to defective mitophagy (Fang et al., 2019). This evidence indicates that different types of mitochondrial dysfunction occur in NDDs. Although it is still unknown whether mitochondrial dysfunction is the cause or functional outcome of NDDs, reversing mitochondrial dysfunction can be an effective means of treating the disease (Burtscher et al., 2022). IPSC modeling shows great potential in figuring out the occurrence of mitochondrial dysfunction in NDDs. In this review, we summarize several differentiation methods that induce iPSCs into characteristic neurons for modeling NDDs such as AD, PD, and ALS. We also recapitulated the mitochondrial abnormalities in differentiated neurons including mitochondrial dynamics and transport, mitochondrial reactive oxygen species (ROS) and mitophagy, mitochondrial energy metabolism dysfunction, and treatment approaches targeting mitochondrial abnormalities.

## 2 IPSC differentiation protocols in neurodegenerative diseases

AD, a common neurodegenerative disease, is the major cause of dementia (Ayodele et al., 2021). The neuropathology of AD is characterized by the progressive accumulation of parenchymal amyloid-β (Aβ) and by the hyperphosphorylation and aggregation of tau protein into neurofibrillary tangles (Venkatramani and Panda, 2019; Pardo-Moreno et al., 2022). Thus, researchers have differentiated hiPSCs into specific neurons, including neural stem cells, cortical neurons, and astrocytes, to explore the pathological changes in AD (Emdad et al., 2011; Krencik and Zhang, 2011; Roybon et al., 2013; Tcw et al., 2017). Dual SMAD signaling was inhibited by SB431542 (SB, an inhibitor of activin-nodal signaling) and LDN193189 (LDN, an inhibitor of BMP signaling) at the first neural induction step (Chambers et al., 2009), following which several small-molecule compounds were used to obtain cortical tissue. For instance, Neurobasal and B27 supplements replaced NPC culture medium at day 10 and were supplemented with 20 ng/ml brain-derived neurotrophic factor (BDNF) to induce final neural maturation at day 30 (Martín-Maestro et al., 2017). For astrocyte differentiation, after the NPCs were obtained, the cells were cultured in suspension with an astrocyte differentiation medium supplemented with basic fibroblast growth factor (bFGF or FGF2) and epidermal growth factor (EGF). The spheres were maintained in suspension for 5–7 months, after which they were associated and plated on Matrigel-coated dishes using ciliary neurotrophic factor (CNTF) and bone morphogenetic protein 4 (BMP4) for maturation (Oksanen et al., 2017). Because the differentiation process of astrocytes can take months, some innovative techniques have been created. Li et al. found that CRISPR‒Cas9-mediated expression of the transcription factors nuclear factor I A (NFIA) or NFIA plus SRY-box transcription factor 9 (SOX9) enables the rapid acquisition of astrocytes from hPSCs within 4–7 weeks, which undoubtedly speeds up the iPSC-derived astrocyte modeling of diseases (Li X. et al., 2018).

PD is the second most common neurodegenerative disease. It is predicted that by 2040, there will be 14.2 million persons with PD, up from 6.9 million in 2015 (Dorsey and Bloem, 2018). Patients diagnosed with PD always have movement and physical problems, such as tremors, stiffness, slowness, and imbalance (Armstrong and Okun, 2020). The pathological hallmark of PD is the Lewy body consisting largely of α-synuclein protein aggregations and loss of dopaminergic neurons in the substantia nigra (Armstrong and Okun, 2020). The floor plate (FP) cells are located at the ventral midline of the neural tube and are the source of midbrain dopamine neurons (DA neurons) (Placzek and Briscoe, 2005; Ono et al., 2007). The protocols to obtain DA neurons are also being modified (Cooper et al., 2010; Kriks et al., 2011; Xi et al., 2012; Wang et al., 2015; Kim et al., 2021). Christopher A. Fasano induced hiPSC to FP cells using noggin with SB431542 to inhibit dual SMAD signaling and Sonic C25II to activate sonic hedgehog (SHH) signaling; treatment with FGF8 or Wnt1 led to a conversion of FP identity into a caudal, midbrain-like identity (Fasano et al., 2010). Subsequently, Kriks et al. demonstrated that the simultaneous use of LSB (LSN + SB)/FGF8/CHIR (GSK3 inhibitor) increases the yield of FOXA2^+^/TH^+^ expressing DA neurons from FP cells, and the DA neurons generated with their protocol possess the essential physiological traits of mature substantia nigra pars compacta DA neurons and the viability of transplantation to the animal models (Kriks et al., 2011). Modification of CHIR concentration to a narrow range was reported to play an important role in inducing DA neurons from iPSC (Xi et al., 2012). They used 0.4 uM CHIR and 500 ng/ml SHH to treat iPSC for 12 days, and then added 20 ng/ml SHH with 100 ng/ml FGF8 at day 13–28, resulting in TH^+^/En1^+^ co-expressing DA neurons.

ALS is a fatal neurodegenerative disease that causes selective degeneration of motor neurons (MNs) in the motor cortex, brainstem, and spinal cord (Vandoorne et al., 2019). ALS patients always suffer from progressive muscle weakness and respiratory failure and ultimately die within 3–5 years (Brown and Al-Chalabi, 2017). Since MNs were used to study the pathology of ALS in recent years, the differentiation protocol of IPSCs to MNs is constantly being refined (Hu and Zhang, 2009; Egawa et al., 2012; Qu et al., 2014; Du et al., 2015; Dafinca et al., 2020). In 2006, Hu and Zhang published a protocol that can generate more than 50% of HB9-expressing motor neurons within 5 weeks. They induced iPSCs into neuroepithelial (NE) with a neural differentiation medium including instantly added cAMP, ascorbic acid, BDNF, glial-derived neurotrophic factor (GDNF), and insulin-like growth factor-1 (IGF1). The authors used 100 nM retinoic acid (RA) on day 10 and 1 µM purmorphamine (targeting smoothened of the SHH signaling pathway, pur) at day 15 to pattern NE cells to ventral spinal progenitor fate, which expresses Olig2. After plating spheres on substrates, several neurotrophic factors and cAMP are added to support the survival and process outgrowth of motor neurons expressing HB9 (Hu and Zhang, 2009). Although later reports added dual inhibition of SMAD signaling and smoothened agonist (SAG) or compound C (AMPK inhibitor) on the basis of a previous protocol (Amoroso et al., 2013; Qu et al., 2014), significantly shortening the differentiation time, the purity of motor neurons still requires improvement. Surprisingly, Du and his colleagues (Du et al., 2015) used a small-molecule cocktail including SB, DMH1 (SMAD inhibitor), CHIR, RA, and pur to obtain a near-pure population of OLIG2^+^ MNPs in 12 days. Then, they added compound E (a NOTCH inhibitor) during MN maturation to improve the purity of motor neurons, resulting in the generation of highly pure (>90%) motor neurons expressing CHAT in 16 days. Because of the shorter time for differentiation into highly pure motor neurons, the protocol has been widely applied in the disease modeling research (Ding et al., 2022; Liu et al., 2022).

AD, PD, and ALS involve corresponding neurons, thereby accurate differentiation of iPSC is significant for research. For instance, mature dopamine neuron subtypes co-expressing TH, NR4A2, and FOXA2 have different gene expression profiles and have differential responses to oxidative stress (Fernandes et al., 2020), emphasizing that there remains a need for referring to the protocol established by other laboratories using small chemical molecules to generate more mature and specific neurons. Table 1 summarizes the mainstream protocols of astrocytes, DA neurons, and MNs differentiation. The most widely applied protocols were featured as less time-consuming and with high purity. The cost of small molecule compounds applied in the protocol also needs to be considered.

**TABLE 1:** Protocols of Astrocytes, DA neurons and MNs differentiation.

Cell type	EGF	FGF2	CNTF	Days	Purity	reference
Astrocytes	D21-D90 10 ng/ml	D21-D90 10 ng/ml	D84-D90 10 ng/ml	90–120	>90% S100β/GFAP^+^	Krencik and Zhang, (2011)
	Day6-12 20 ng/ml	Day6-9 10 ng/ml; (FGF)	Day9-35 20 ng/ml	35	78% GFAP^+^	Emdad et al. (2011)
		Day9-15 20 ng/ml				
	Day28–35	Day28–35 -; Day90-97 50 ng/ml	Day15-31	100	∼100% S100β^+^	Roybon et al. (2013)
			—			
	—	Day15-45 20 ng/ml	—	45	90% S100β^+^	Tcw et al. (2017)
					82% GFAP^+^	
	CHIR	SHH	FGF8	Days	purity	References
DA neurons	-	Day12-42 100 ng/ml	Day12-42 100 ng/ml	49	<1% TH^+^/β-tubulin/FOXA2^+^	Cooper et al. (2010)
	Day3-13 3uM	Day1-7 100 ng/ml	Day 1–7 100 ng/ml	50	70%-80%TH^+^	Kriks et al. (2011)
					50% Nurr1^+^	
	Day1-11 0.4 uM	Day1-11 500 ng/ml	Day12-28 100 ng/ml	35	43.6 ± 6.2% cells TH^+^, which co-expressed Nurr1 (95.3 ± 2.4%), En1 (96.2 ± 1.1%)	Xi et al. (2012)
		Day12-28 20 ng/ml				
	Day1-6 1 uM	Day7-18 100 ng/ml	Day7-18 100 ng/ml	32	10.5% TH^+^/GIRK2^+^	Wang et al. (2015)
	Day0-4 0.7 uM	Day0-7 500 ng/ml	-	30	-	Kim et al. (2021)
	Day4-10 7.5 uM					
	Day10-11 3 uM					
	CHIR	Compound C	SHH	Days	purity	References
MNs	—	—	Day15-28 100 ng/ml	35	∼50% HB9 ^+^	Hu and Zhang, (2009)
			Day28-35 50 ng/ml			
	—	Day0-12 2 μM	Day12-22 100–500 ng/ml	35	—	Egawa et al. (2012)
	—	Day0-6 1 μM	Day6-20 100 ng/ml	24	69.5% HB9^+^	Qu et al. (2014)
	Day0-6 3 μM	—	(purmophamine)	28	90% HB9^+^	Du et al. (2015)
	Day6-12 1 μM		Day6-12 0.5 μM		95% ISL1^+^	
			Day12-18 0.1 μM		91% CHAT^+^	
	Day0-6 3 μM	Day0-6 1 μM	Day2-19 500 ng/ml	30	90% Tuji1^+^	Dafinca et al. (2020)
					90% CHAT^+^	

FGF, epidermal growth factor; FGF2/8, Fibroblast growth factor2/8; CNTF, ciliary neurotrophic factor; CHIR, GSK3 inhibitor; SHH, sonic hedgehog agonist; Compound C, AMPK, inhibitor.

## 3 IPSC modeling in neurodegenerative diseases

AD is divided into familial AD (FAD) and sporadic AD (sAD), and FAD constitutes less than 3% of all AD subtypes. The APP gene and the proteolytic enzymes that produce peptide A, presenilin 1 and 2, are the two primary genes that are mutated in FAD (García-Morales et al., 2021). IPSC modeling demonstrates that there are some common pathological features in FAD and sAD (Bélanger et al., 2011; Piers et al., 2020). Jones et al. induced astrocytes derived from AD iPSC have been reported to display cellular atrophy and to have aberrant expression and localization of S100B, EAAT1 and GS, which are cell-autonomous instead of compromised neuronal intermediates, supporting the conjecture that astrocyte dysfunction is an early hallmark of AD (Jones et al., 2017). Moreover, iPSC modeling provides a comprehensive phenotype study of AD. PSEN1 iPSC-derived astrocytes displayed robust accumulation of full-length presenilin-1, increased secretion and compromised uptake of Aβ1–42, disturbed Ca^2+^ signaling, and altered metabolism (Oksanen et al., 2017). In addition, astrocytes derived from AD iPSC co-cultured with healthy astrocytes provide an opportunity to observe the pathological microenvironment, including the effects of AD astrocytes and autonomic response of healthy astrocytes.

Likewise, SNCA (α-synuclein), PARK2 (parkin), PARK7 (DJ-1), PINK1 and LRRK2 are the mutated genes of parkinsonism, among which mutations in both PARK2 (parkin) and LRRK2 are the most common genetic causes (Healy et al., 2008). However, the cause of PD remains unclear at the molecular level. IPSC modeling of mutant genes, including PARK2 and LRRK2, helps to investigate PD occurrence and development, and can target a pathway to delay disease progression (Cooper et al., 2012; Wasner et al., 2022). In PD iPSC-derived DA neurons, Jian Feng found higher protein level of tyrosine hydroxylase (TH) and high ROS levels. Moreover, there is no significant alterations in DA levels, which might be due to homeostatic regulation (Ren et al., 2022). However, decreased TH mRNA level and DA release were reported in postmortem brain tissues and cerebrospinal fluid of PD patients respectively (Goldstein et al., 2012; Zhang et al., 2022). The inconsistent phenotype between iPSC modeling and postmortem tissues may precisely account for the transition from early to late stage of diseases. Young-onset PD iPSC-derived DA neurons displayed increased accumulation of soluble α-synuclein protein and phosphorylated protein kinase Cα, as well as dysregulated of lysosomal biogenesis and function, establishing a highly predictive phenotype of young-onset PD (Laperle et al., 2020).

ALS familial ALS (fALS) and sporadic ALS (sALS). In fALS, mutations in the “superoxide dismutase 1” (SOD1) gene are the most popular genetic factor, and the remaining common mutation genes are “fused in sarcoma” (FUS), “TAR DNA binding protein” (TARDBP), or a hexanucleotide repeat expansion in the “chromosome nine open reading frame 72” (C9ORF72) (Taylor et al., 2016). ALS-related iPSC modeling is able to reveal the sequence of its occurrence and development and provide a platform to explore the molecular mechanism of its pathology (Chen et al., 2014; Kiskinis et al., 2014; Lopez-Gonzalez et al., 2016; Dafinca et al., 2020; Altman et al., 2021). Chen et al. generated MNs and non-MNs derived from ALS SOD1 iPSC to reveal that neurofilament aggregation is an early event in ALS pathogenesis, resulting from the dysregulation of NF subunit proportion caused by SOD1 mutant binding NF-L mRNA in MNs (Chen et al., 2014). Likewise, iPSC modeling undoubtedly provides a good research tool for studying of sporadic ALS (Burkhardt et al., 2013; Fujimori et al., 2018a; Fujimori et al., 2018b; Coyne et al., 2021). Moreover, it has been found that intranuclear TDP-43 aggregation in iPSC-derived MNs reprogrammed from three sALS patients and firstly validated it in postmortem tissue from one of the previous sALS patients, which highlights the importance of iPSC modeling in sALS (Burkhardt et al., 2013).

## 4 Advantages and disadvantages of iPSC modeling

IPSC modeling of different phenotypes using mature protocols showcased stability and scalability, which together with its high yield facilitates high-throughput screening of clinical drugs (Pasteuning-Vuhman et al., 2021). In addition, iPSC modeling overcame the problem of clinical drug screening caused by heterogeneity of NDDs patients. Okano et al. used two types of MNs carrying FUS and TDP43 to screen 1,232 drugs. They ultimately selected ropinirole as the candidate, which is ineffective on the phenotypes of SOD1-mutant iPSC-derived MNs (Fujimori et al., 2018a). Thus, IPSC-derived neurons carrying patients’ specific genetic background help to identify drug responders, contributing to patient stratification in clinical studies (Haston and Finkbeiner, 2016).

In addition, iPSC-based disease modeling *in vitro* is different from animal models, including the neuronal physiological morphology and function and the molecular characteristics of pathological states. In AD, human protoplasmic astrocytes are bigger and more complex than their rodent counterparts, and the calcium wave in mouse astrocytes transmitted much more slowly than in human astrocytes (Oberheim et al., 2009). Chen et al. reported that there was no SOD1 aggregation or mitochondrial swelling in human ALS MNs but these appeared in SOD1 transgenic mice (Chen et al., 2014). Most importantly, mouse models fail to mimic sporadic neurodegenerative diseases, which account for most NDDs (Hawrot et al., 2020). However, mouse models are advantageous because they exhibit complex interactions between cells and the microenvironment, as well as provide behavioral assessment and pharmacokinetics features (Ke et al., 2021). Therefore, a promising way with the combination of advantages in both iPSC modeling and mouse models would contribute to a complementary and comprehensive view of NDD researches.

There are still some limitations for iPSC remodeling NDDs, such as the lack of aging-signature. Lorenz Studer and his lab overexpressed progeria with the treatment of modified-RNAs in PD iPSC-derived DA neurons, and found a significant reduction in dendrite length and downregulation of AKT signaling (Miller et al., 2013). Moreover, chemical pretreatment also accelerates the maturation of neurons (Fujimori et al., 2018a). Thus, progerin-induction or chemical pretreatment combing with iPSC-modeling overcomes the problem of iPSCs taking a long time to naturally develop into the degenerative disease stage, which mainly includes the expression of aging signatures. Another modeling method is direct neuronal conversion to induced neurons (iNs), which maintains the aging signature and epigenetic status of neurons (Vierbuchen et al., 2010; Mertens et al., 2015). However, the high cost and low cell output limit its application; future improvements should address these issues.

## 5 Normal mitochondrial function

**FIGURE 1:**
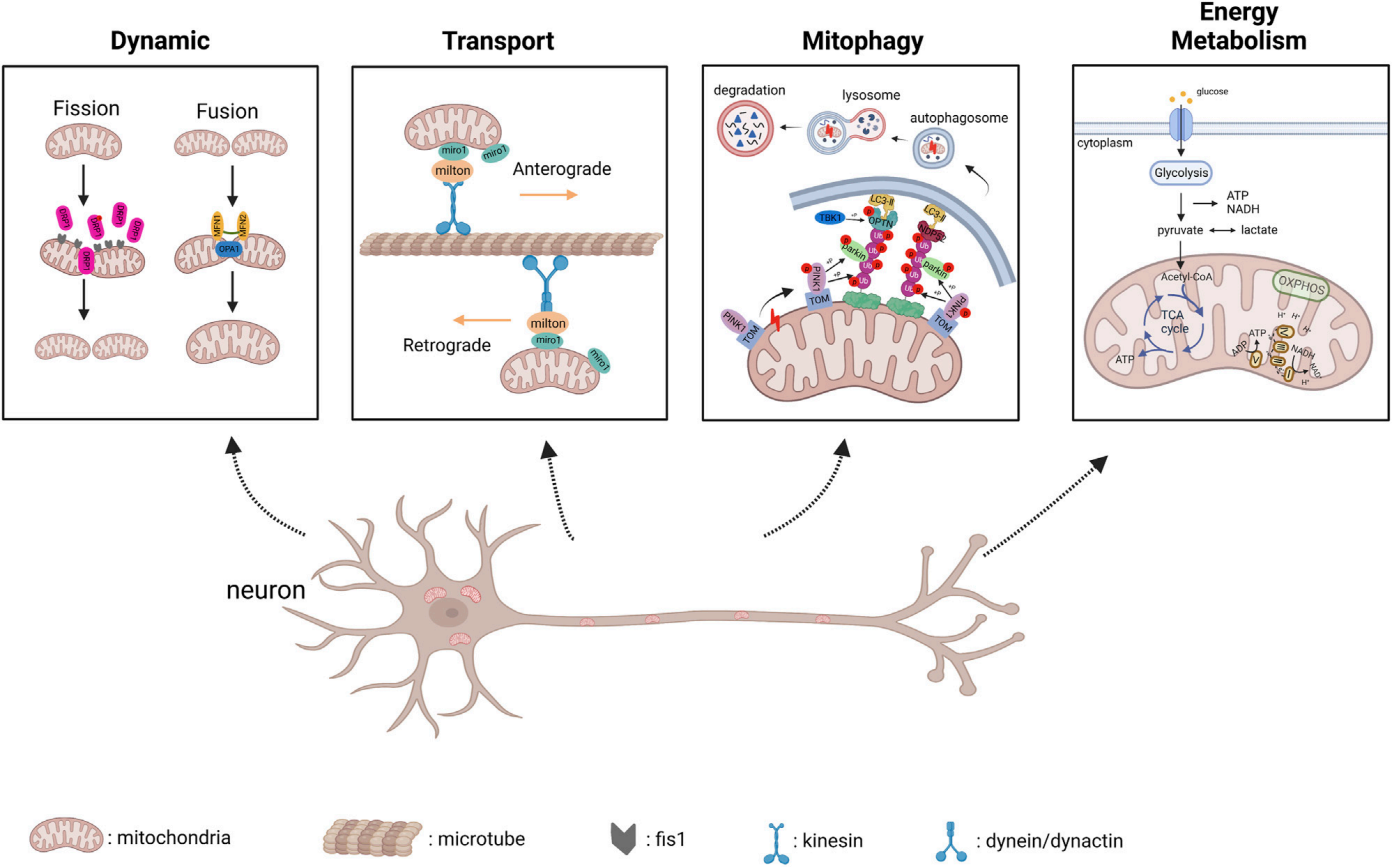
Physiological progress of mitochondrial dynamics, transport, PINK1/parkin-mediated mitophagy and energy metabolism (created with BioRender.com). 1) Dynamic: DRP1 is recruited to mitochondrial membrane along with Fis1 to regulate mitochondrial fission; MFN1/2 mediates fusion of mitochondrial outer membrane and OPA1 mediates fusion of inter membrane. 2) Transport: Anterograde transport is mediated by kinesin motor and Miro/Milton adaptors, and retrograde transport is mediated by dynein/dynactin and Miro/Milton adaptors. 3) PINK1/parkin-mediated Mitophagy: Once mitophagy starts, PINK1 accumulates in mitochondria, and in turn results in PINK1 autophosphorylation, which subsequently triggers ubiquitin phosphorylation, and recruits parkin to damaged mitochondria; Parkin ubiquitinates mitochondrial outer membrane proteins, and Optineurin interacts with these proteins tagged by ubiquitin and targets the isolation membrane to damaged mitochondria, which are eventually sent to lysosomes for degradation. 4) Energy Metabolism: Glycolysis occurs in cytoplasm, and produce ATP, NADH as by-product; The pyruvate molecules derived from glycolysis are imported to mitochondria and generate acetyl-CoA molecules, which will enter the tricarboxylic acid (TCA) cycle. Finally, OXPHOS complexes located in mitochondrial inter membrane uses NADH and FADH2 to generate ATP.

IPSC modeling has undoubtedly greatly contributed to the knowledge of degenerative diseases, including mitochondrial dysfunction. Healthy mitochondria can maintain their own homeostasis through mitochondrial dynamics and autophagy even in the face of external stimuli, thereby exerting stable functions (Figure 1).

### 5.1 Mitochondrial dynamics and transport

Mitochondria continuously undergo fusion and fission, which are known as mitochondrial dynamics, to exchange metabolites (Schrepfer and Scorrano, 2016). MFN1 (membrane protein mitofusin 1) and MFN2 (membrane protein mitofusin 2) are fusion-related proteins, while DRP1 (dynamin-related protein 1) and Fis1 (mitochondrial fission one protein) are responsible for mitochondrial fission. Mitochondrial transport between Soma and distal axonal was mediated by motor proteins kinesin and dynein, which correspond to anterograde and retrograde transport respectively (Lin and Sheng, 2015). Moreover, miro and Milton serve as motor adaptors involved in mitochondrial transport (Lin and Sheng, 2015). It is reported that defective mitochondrial transport has been associated with NDDs (Cozzolino et al., 2013; Cai and Tammineni, 2017; Prots et al., 2018).

### 5.2 Mitochondrial ROS and mitophagy

Reactive oxygen species (ROS) include free radicals such as superoxide anion (O2^−^), hydroxyl radical (HO^−^), and non-radical hydrogen peroxide (H_2_O_2_), which are the endogenous sources of a mitochondrial respiratory chain under the high demand of neurons for oxygen and ATP (Chen et al., 2012). There is a hypothesis that mutant SOD1 has the ability to generate HO^−^ from H2O2, thus creating an oxidative environment and toxic aggregates in MNs (Julien, 2001). As for PINK1/parkin-mediated mitophagy, When the mitochondrial membrane potential decreases, PINK1 accumulates in mitochondria and gets autophosphorylation, which subsequently triggers ubiquitin phosphorylation, and recruits parkin to damaged mitochondria. Subsequently, Parkin ubiquitinates mitochondrial outer membrane proteins and Optineurin interacts with these proteins tagged by ubiquitin and targets the isolation membrane to damaged mitochondria, which are eventually sent to lysosomes for degradation (Wei et al., 2015; Rasool et al., 2018; Goiran et al., 2022; Pradeepkiran et al., 2022).

### 5.3 Mitochondrial energy metabolism

Neurons in the brain need energy expenditure to maintain various functional activities, including synaptic transmission processes (Faria-Pereira and Morais, 2022). The major metabolic precursors for biosynthesis and energy generation come from the TCA cycle and glycolysis (Zheng et al., 2016). The pyruvate molecules produced in cytosolic glycolysis can be imported into the mitochondria and generate acetyl-CoA molecules, which will enter the tricarboxylic acid (TCA) cycle. Finally, OXPHOS complexes use NADH and FADH2 to generate ATP from this cycle (Faria-Pereira and Morais, 2022). Neurons tend to rely on mitochondrial OXPHOS to meet their energy demands, thereby dysfunction of this process would cause NDDs (Breuer et al., 2013; Koopman et al., 2013).

## 6 Mitochondrial dysfunction and therapeutic strategies in iPSC modeling

It is widely recognized that neurodegenerative diseases are closely related to mitochondrial dysfunction (Wang et al., 2009; Queliconi et al., 2021; Mehta et al., 2022). Just as the same disease may manifest in different ways, so the same disease modeling will also have different representations of mitochondrial dysfunction. Tables 2–4 summarizes the iPSC-based modeling of AD, PD, ALS, and the associated characterization of mitochondrial dysfunction. As for neurons derived from AD or PD iPSC, they mostly exhibit impaired mitophagy and dysregulated mitochondrial dynamics including increased mitochondrial fragments, while aberrant mitochondrial transport and reduced MMP are more common in ALS iPSC derived MNs (Dafinca et al., 2016; Zanon et al., 2017; Naumann et al., 2018; Martín-Maestro et al., 2019; Li et al., 2020). Also, PD iPSC modeling has shown abnormal mitochondrial morphology and increased ROS level (Imaizumi et al., 2012; Shaltouki et al., 2015; Chung et al., 2016). Furthermore, neurons from iPSC of NDDs manifest abnormal energy metabolism, showing decreased basal glycolysis and ATP level in AD iPSC modeling (Bélanger et al., 2011; Konttinen et al., 2019), reduced of complex I activity and decreased basal respiration in PD iPSC modeling (Zanon et al., 2017; Zambon et al., 2019), as well as impaired basal respiration and increased glycolysis in ALS iPSC modeling (Hor et al., 2021; Mehta et al., 2021). Sometimes neurons carrying the same mutant gene displayed different even contradictory energy metabolism, which may be attributed to the culture state and sensible to testing environment. Moreover, these pathological mechanisms are interconnected. Many studies have confirmed that the mitochondrial fission and fusion machinery is coupled with mitochondrial transport and mitophagy (Misko et al., 2010; Wu et al., 2016; Gao et al., 2017). Mitophagy regulates the mitochondrial energetic status in neurons (Han et al., 2021), thus damage to any link may lead to the occurrence and development of NDDs. Next, we describe mitochondrial dysfunctions of mitochondrial dynamic, transport, mitophagy, and energy metabolism abnormalities in AD, PD, ALS, and their corresponding treatment strategies.

**TABLE 2:** Mitochondrial dysfunction in iPSC-derived neurons and astrocytes modeling of AD.

Type	Neuron	Mutated gene	Mitochondrial dysfunction	References
fAD	Astrocytes	PSEN1	Increased cellular ROS level	Oksanen et al. (2017)
fAD	Cortical neuron	PSEN1	Impaired axonal transport of mitochondria and the balance of mitochondrial fusion and fission	Li et al. (2020)
fAD	Cortical neuron	PSEN1	Impaired mitochondria fission and fusion protein levels	Li et al. (2018a)
sAD	Microglia	TREM2	Impaired mitochondrial respiration, enhanced mitochondrial superoxide levels	Piers et al. (2020)
fAD	Neural stem cell	PSEN1	Impaired mitophagy, Impaired balance of mitochondrial fission and fusion	Martín-Maestro et al. (2019)
fAD, sAD	Cortical neuron	APOE4	Defective mitophagy, compromised mitochondrial homeostasis, increased mitochondrial fragmentation, low ATP levels	Fang et al. (2019)
		APP		
fAD	Astrocytes	PSEN1	Increased basal respiration, decreased basal glycolysis and lactate	Bélanger et al. (2011)
fAD	Astrocytes	PSEN1	Decreased basal glycolysis and impaired fatty acid oxidation	Konttinen et al. (2019)
fAD	Astrocytes	APOE	Increased mitochondrial respiration and ATP production, elevated glycolytic activity	Ryu et al. (2021)

**TABLE 3:** Mitochondrial dysfunction in iPSC-derived DA neuron modeling of PD.

Type	Mutated gene	Mitochondrial dysfunction	References
fPD	PINK1	Reduced levels of basal respiration, disrupted mitochondrial movement	Cooper et al. (2012)
	LRRK2		
fPD	PARK2	Reduction of complex I activity, fragmented mitochondria	Zanon et al. (2017)
fPD	PINK1	Impaired mitophagy	Oh et al. (2017)
fPD	SNCA	Delayed mitophagy	Shaltouki et al. (2018)
fPD	PARK14	Increased ROS level, decreased MMP, dominant mitochondrial fragmentation	Ke et al. (2020)
fPD	PARK2	Increased cellular ROS, Abnormal mitochondrial morphology and impaired mitochondrial turnover, aberrant degradation of damaged mitochondria	Imaizumi et al. (2012)
fPD	PARK2	Abnormal mitochondrial morphology, Increased mitochondrial ROS	Chung et al. (2016)
	PINK1		
fPD	PARK2	Decreased mitochondrial abundance, abnormal mitochondrial morphology	Shaltouki et al. (2015)
fPD	SNCA	Abnormal mitochondrial morphology, reduced MMP, decreased basal respiration and ATP production, and decreased lactate production	Zambon et al. (2019)
fPD	PARK2	Decreased protein level of cytochrome c oxidase (COX) IV and mitochondrial dynamics protein MEF1	Bogetofte et al. (2019)
fPD	PARK2	Reduced MMP, disrupt mitochondrial ultrastructure, and impaired mitochondrial respiration	Okarmus et al. (2021)
fPD	PARK2	reduced mtDNA levels of subunits of the electron transport chain (ETC)	Wasner et al. (2022)
fPD	LRRK2	Delayed axon mitophagy, reduced mitochondrial motility	Hsieh et al. (2016)
fPD	LRRK2	Decreased MMP, increased ROS, mitochondrial fragmentation	Su and Qi, (2013)
fPD	PINK1	Reduced levels of ATP production	Vos et al. (2017)

**TABLE 4:** Mitochondrial dysfunction in iPSC-derived MNs modeling of ALS.

Type	Mutated gene	Mitochondrial dysfunction	References
fALS	C9ORF72	Increased ROS level	Lopez-Gonzalez et al. (2016)
fALS	FUS	Stalled and short mitochondrion, lost membrane potential on distal axon	Naumann et al. (2018)
sALS	C9orf72	Reduced MMP, abnormal mitochondrial morphology	Dafinca et al. (2016)
fALS	FUS	Reduced number of moving mitochondria	Guo et al. (2017)
fALS	A4V	More vacuolar mitochondria, reduced number of moving mitochondria	Kiskinis et al. (2014)
fALS	C9ORF72 TARDBP	Impaired mitochondrial Ca2^+^ buffering	Dafinca et al. (2020)
fALS	TARDBP	Reduced number of moving mitochondria	Fazal et al. (2021)
fALS	TARDBP	Reduced mitochondrial protein synthesis	Altman et al. (2021)
fALS	SOD1	Reduced MMP, increased mean length of mitochondria, Reduced ATP Levels	Günther et al. (2022)
fALS	TARDBP	Mitochondrial fragmentation	Choi et al. (2020)
fALS	C9orf72	Impaired basal respiration	Mehta et al. (2021)
fALS	SOD1 TARDBP C9ORF72; sALS1	Reduced basal respiration and ATP production, increased glycolysis and lactate	Hor et al. (2021)
sALS	sALS2		
	sALS3		

### 6.1 Alzheimer’s disease

In AD, a study reported that the balance of mitochondrial fusion and fission was disrupted in PSEN1-E120K iPSC-derived cortical neurons, with an increase in DRP1 and a decrease in MFN1 (Li L. et al., 2018). After 2 years, this team found the same phenomenon in PSEN1-S170F iPSC-derived cortical neurons (Li et al., 2020). Aβ and phosphorylated tau interact with DRP1, which activates DRP1 by different pathways, causing impairments in mitochondrial dynamics (Manczak et al., 2011; Kandimalla et al., 2016). Since Aβ is produced by the sequential cleavage of APP *via ß*-site APP cleaving enzyme 1 (BACE1), so γ-secretase, an inhibitor of BACE1 (BSI and 5 μmol/L LY2884721), significantly reduced the levels of Aβ and p-Tau (Li et al., 2020). However, Fang explained that defective mitophagy induces AMPK activation (p-AMPK), which leads to excessive mitochondrial fragmentation. They used two mitophagy detection nematode lines to screen the enhancer for mitophagy, which ameliorated neuronal pathology and mitochondrial fragments in AD iPSC-derived cortical neurons (Fang et al., 2019).

Mitophagy plays an important role in maintaining cell homeostasis, including the removal of damaged mitochondria and resistance to oxidative stress (Scheibye-Knudsen et al., 2015). Martín-Maestro et al. demonstrated dysregulation of mitophagy because of deficient lysosomal function in iPSC-derived cortical neurons from FAD1 patients harboring PSEN1 A246E mutation (Martín-Maestro et al., 2017). After 2 years, they found the same problem in PSEN1 M146L iPSC-derived NSCs, along with the downregulation of oxidative phosphorylation (OXPHOS)-related proteins, suggesting an impaired mitochondrial respiratory chain (Martín-Maestro et al., 2019). Deficiency in autophagy induction and lysosomal acidification was confirmed as the main reason for mitophagy failure, and that was corrected by using bexarotene, an FDA-approved retinoid X receptor (RXR) agonist, whose therapeutic efficacy stems from increased mitophagy flux (Martín-Maestro et al., 2019). Another study reported that the abnormal accumulation of Aβ and p-Tau decreases the levels of activated mitophagy proteins in AD iPSC-derived cortical neurons, leading to the accumulation of damaged mitochondria and the elevation of ROS levels (Fang et al., 2019). The authors screened two mitophagy inducers (urolithin A and actinonin) to restore mitophagy, further improving mitochondrial function by reducing mitochondrial ROS and ameliorating AD’s pathology. As mentioned above, treatments targeting impaired mitophagy display a significant role in rescuing neuronal apoptosis (Shaltouki et al., 2018; Fang et al., 2019). In addition, Kshirsagar et al. found that several mitophagy enhancers, especially UA were able to rescue mitochondrial dysfunction and increase cell survival in AD HT22 cell models, indicating that mitophagy enhancers could developed as drugs to delay neurodegenerative pathology in clinical patients (Kshirsagar et al., 2021, 2022).

With regard to energy metabolism, Minna Oksanen et al. reported increased basal respiration and decreased basal glycolysis in PSEN1 iPSC-derived astrocytes, as typical astrocytes rely more on glycolysis (Bélanger et al., 2011). Additionally, the authors detected more cellular ROS and less lactate secretion in these astrocytes, suggesting that the energy metabolism activity of the PSEN1 mutation made a switch from glycolysis to OXPHOS (Oksanen et al., 2017). Given that neurons may sustain themselves with lactate generated by glycolysis, this alteration may not be advantageous to astrocytes (Figley, 2011). Similarly, another study conducted by the same laboratory reported impaired fatty acid oxidation (FAO) except for the same changes in basal respiration and basal glycolysis in PSEN1 iPSC-derived astrocytes, and using a PPARβ/δ-agonist is beneficial for impaired FAO and memory deficits in APP/PSEN1 mice (Konttinen et al., 2019). Ryu et al. published a more specific metabolism study of AD astrocytes and NPC. Their results showed increased mitochondrial respiration, energy (ATP) output, and elevated glycolytic activity, which are partly different from the results of previous studies. Considering the reduced ability to absorb glucose, as well as the deficiencies in the generation and transfer of reducing agents throughout the glycolytic process and the mitochondrial respiratory chain, upregulating OXPHOS and glycolysis is the way for AD astrocytes and NPCs overcompensate. Moreover, the levels of NAD^+^ and NADH are significantly decreased in AD astrocytes in NPC, despite the general biochemical reducing power in LOAD not being compromised, which reflects aberrant energy metabolic activity in AD astrocytes and NPC(Ryu et al., 2021).

### 6.2 Parkinson’s disease

Regarding PD, its main mutated genes, PARK2 and PINK1, are involved in mitochondrial functions, and there is no doubt that mitochondrial dysfunction is one of the pathological features of Zanon reported that DA neurons derived from the PARK2 mutation carrier iPS-B125 showed fragmented mitochondria, which was compensated by SLP-2 overexpression (Zanon et al., 2017). Parkin and SLP-2 are able to interact functionally to maintain mitochondrial function (Zanon et al., 2017). Ke et al. detected upregulation of the fission proteins DRP1 and Fis1 and downregulation of the fusion protein MFN1 in PLA2G6 mutant DA neurons (Ke et al., 2020). The PLA2G6 mutant induced ER stress, which facilitated the unfolded protein response (UPR), resulting in decreased signaling of CREB and final mitochondrial fragments. Thus, they used azoramide (a small-molecule modulator) to enhance the CERB signaling, and rescue mitochondrial function, thereby preventing the apoptosis of DA neurons (Ke et al., 2020). Given that the PD-associated LRRK2 mutant interacts with DRP1 to recruit and phosphorylate mitochondrial DRP1 (Wang et al., 2012), it is not surprising that Su and Qi found increased mitochondrial fragmentation in LRRK2 G2019S-derived DA neurons, and used P110 to inhibit the hyperactivation of DRP1 to reduce mitochondrial fragments (Su and Qi, 2013).

Mitophagy is closely related to the pathological mechanism of PD. Aberrant elimination of mitochondria treated with carbonyl cyanide m-chlorophenyl hydrazine (CCCP) was reported in PARK2 iPSC-derived neurons (Imaizumi et al., 2012). Mitophagy plays an important role in maintaining cell homeostasis, including the removal of damaged mitochondria and resistance to oxidative stress (Scheibye-Knudsen et al., 2015). Yu-Chin Su and colleagues detected increased mitochondrial fragmentation, which resulted in a defective ETC complex and an elevated level of mitochondrial ROS by DRP1 hyperactivation in LRRK2 G2019S DA neurons (Su and Qi, 2013). Subsequently, increased mitochondrial fragmentation simulates excessive mitophagy, which causes a loss of mitochondrial mass. This mitochondrial dysfunction was solved by P110 treatment which inhibits DRP1 hyperactivation (Su and Qi, 2013). Some studies focusing on PARK2 and PINK1 iPSC-derived DA neurons respectively reported higher mitochondrial ROS levels and defective mitophagy (Chung et al., 2016; Oh et al., 2017), among which the author found the PINK1(C568A) mutant could be able to mimic PINK1 S-nitrosylated caused by endogenous NOS, and this PINK1(C568A) mutant prevented parkin from translocating to mitochondrial membrane, thus resulting in impaired mitophagy and neuronal death (Oh et al., 2017). Treatment of these cells with l-NAME could be able to ameliorate damaged mitophagy and neuronal death by inhibiting the production of NOS(Oh et al., 2017). Miro, a protein that anchors mitochondria to microtubule motors, should be removed first, then mitochondria stop trafficking and mitophagy starts (Glater et al., 2006; Wang et al., 2011). Shaltouki and Hsieh found that accumulation of miro1 delayed the initiation of mitophagy in both LRRK2 G2019S and SCNA A53T iPSC-derived DA neurons, and decreased the level of miro1 protein by RNAi promoted mitophagy, and further rescued neurodegeneration (Hsieh et al., 2016; Shaltouki et al., 2018).

In PD SNCA iPSC-derived DA neurons, Zambon et al. found a decrease in basal respiration, spare capacity, ATP and lactate production, and no significant differences in glycolytic activity, suggesting deficits in mitochondrial energy metabolism (Zambon et al., 2019). This suggests that the association between α Syn and TOM20 leads to mitochondrial metabolic dysfunction (Di Maio et al., 2016; Zambon et al., 2019). PINK1 mutant iPSC-derived DA neurons were found to have reduced levels of ATP production, which was improved by treatment with cerulenin to rescue PINK1 deficiency (Vos et al., 2017). Fernandes5 reported downregulation of OXPHOS-related gene expression and upregulation of glycolysis-related gene expression under oxidative stress conditions in one type of SNCA DA neurons (Fernandes et al., 2020). PARK2 knockout DA neurons were reported to have reduced basal respiration and ATP production under only lactate respiration, along with decreased glycolysis and accumulation of lactate, which was confirmed by proteomic and metabolomic analysis of dysregulated key factors and enzymes in mitochondrial energy metabolism (Bogetofte et al., 2019; Okarmus et al., 2021).

### 6.3 Amyotrophic lateral sclerosis

Impaired mitochondrial dynamics can also be observed in ALS-related MN. It was reported that activation of PP1 dephosphorylates DRP1 S616, resulting in increased mitochondrial fission in TDP43-derived MNs. PP1 suppression by treatment with I-2 or okadaic acid (OA) significantly inhibited excessive mitochondrial fission (Choi et al., 2020). Interestingly, although there are numerous reports of impaired mitochondrial dynamics in ALS patients and animals, this has been less reported in ALS-iPSC modeling (Wang et al., 2013; Méndez-López et al., 2021). Deficits in mitochondrial transport along the axon are more common in ALS iPSC-derived MNs, and mitochondrial transport plays an important role in maintaining normal functioning of MNs. Whether it is mitochondrial dynamics or axonal transport, its damage has an important impact on neuron survival and disease progression. Thus, therapeutic strategies targeting both can effectively alleviate disease progression. A study by Guo and colleagues reported that there were fewer moving mitochondria than stationary mitochondria, while the total number of mitochondria remained unaltered in mutant FUS MNs (Guo et al., 2017). The same phenomenon was found in mutant TDP43 MNs (Fazal et al., 2021). However, it remains unclear why mitochondrial transport is impaired in mutated gene-derived MNs. One inference is that cytosolic aggregation or aberrant TDP-43 causes a reduction in mitochondria-associated ER membranes (MAM), and the mitochondrial movement-related protein miro1 preferentially localizes in MAM sites; thus, mitochondrial transport will decrease (Macaskill et al., 2009; Stoica et al., 2014). Interestingly, both different MNs’ mitochondrial transport defects can be solved by HDAC inhibitors, possibly because a rise in the acetylation of α-tubulin increases the number of motor proteins binding to the microtubules, which leads to an increase in mitochondrial transport (Guo et al., 2017).

Surprisingly, mutant forms of proteins (such as optineurin OPTN and TBK1) involved in mediating mitophagy are associated with ALS (Wong and Holzbaur, 2014; Moore and Holzbaur, 2016; Harding et al., 2021); however, there are few articles related to iPSC-based ALS modeling that characterized by defects in mitophagy. Moreover, ALS animals and other cells which transfected the mutated gene of ALS were used to report mitophagy defects (Palomo et al., 2018; Foster et al., 2020). For instance, the mitochondria in N2A cells transfected with SOD1 displayed low levels of mitophagy and high levels of ROS. This can be explained by the fact that mutated SOD1 binds to OPTN and sequesters it in N2A cells, thereby disrupting mitophagy, which in turn leads to increased ROS release (Tak et al., 2020). Overexpression of OPTN reduces the cytotoxicity of mutant SOD1, thus improving mitophagy and decreasing ROS levels (Tak et al., 2020). In addition, there are paths to control mitochondrial quality except for mitophagy (Lin et al., 2017; Gautam et al., 2019). Mukesh Gautam et al. found a unique self-destructive path for mitochondria, and the morphology of defective mitochondria changed including being elongated and curling themselves, then beginning to disintegrate from the inner membrane and the cristae, and finally being eliminated (Gautam et al., 2019). Therefore, whether mitophagy disorder occurs in iPSC-derived MNs carrying mutated ALS-related genes warrants further investigation.

Mitochondrial energy metabolic dysfunction is also a pathologic characteristic of ALS. There are diverse, specialized cell populations in the mammalian brain, and related studies have also suggested that bioenergetic genes and metabolic patterns in different cell types are differentially altered in developing cells or diseases, such as astrocytes and MNs (Qi et al., 2019). Vandoorne et al. reported an interesting finding that when iPSC is differentiated into MNs, its metabolic pattern shifts from glycolytic to oxidative metabolism, along with reduced glycolytic flux and elevated TCA cycle activity (Vandoorne et al., 2019). In addition, the ALS-related FUS mutant does not change the energy metabolism of MNs. Another study focusing on MNs carrying the C9orf72 mutant reported impaired basal and maximal mitochondrial respiration; however, glycolytic function was not affected (Mehta et al., 2021). Moreover, the transcriptomic analysis showed decreased gene expression of the mitochondrial ETC, which was restored by overexpression of PGC1α to stimulate mitochondrial biogenesis and improve mitochondrial function (Mehta et al., 2021). Günther et al. reported that SOD1 mutant iPSC-derived MNs displayed low levels of ATP without other mitochondrial metabolic changes (Günther et al., 2022). Hor and colleagues used three sALS iPSC line-derived MNs and three fALS iPSC line-derived MNs carrying SOD1, TDP43, and C9ORF72 mutants to find reductions in basal respiration, ATP production, and spare respiratory capacity, causing increased glycolysis and lactate. These abnormal changes illustrate impaired mitochondrial respiration and a compensatory response by ALS MNs (Hor et al., 2021). Notably, treatment with C12 ameliorated the mitochondrial energy metabolism *via* SIRT3 activation and mitochondrial deacetylation. The improvement of neuronal mitochondrial energetics through the restoration of OXPHOS and glycolysis as well as the correction of mitochondrial dysfunctions have become increasingly important therapeutic targets for delaying the progression of NDDs (Cunnane et al., 2020).

## 7 Discussion

NDDs are one of the most widespread diseases with a low cure rate, bringing enormous physical pain and economic pressure to patients and a heavy burden to society. At present, our exploration of the NDD is not thorough yet. IPSC modeling provides a panoramic view of the pathophysiology of NDDs. Unlike other models, this type of modeling enables the early-phase mechanism to be uncovered. Moreover, in the era of precise medication, it is a promising method for the personalized identification of unique molecular features in NDDs of either the sporadic or the familiar type.

**FIGURE 2:**
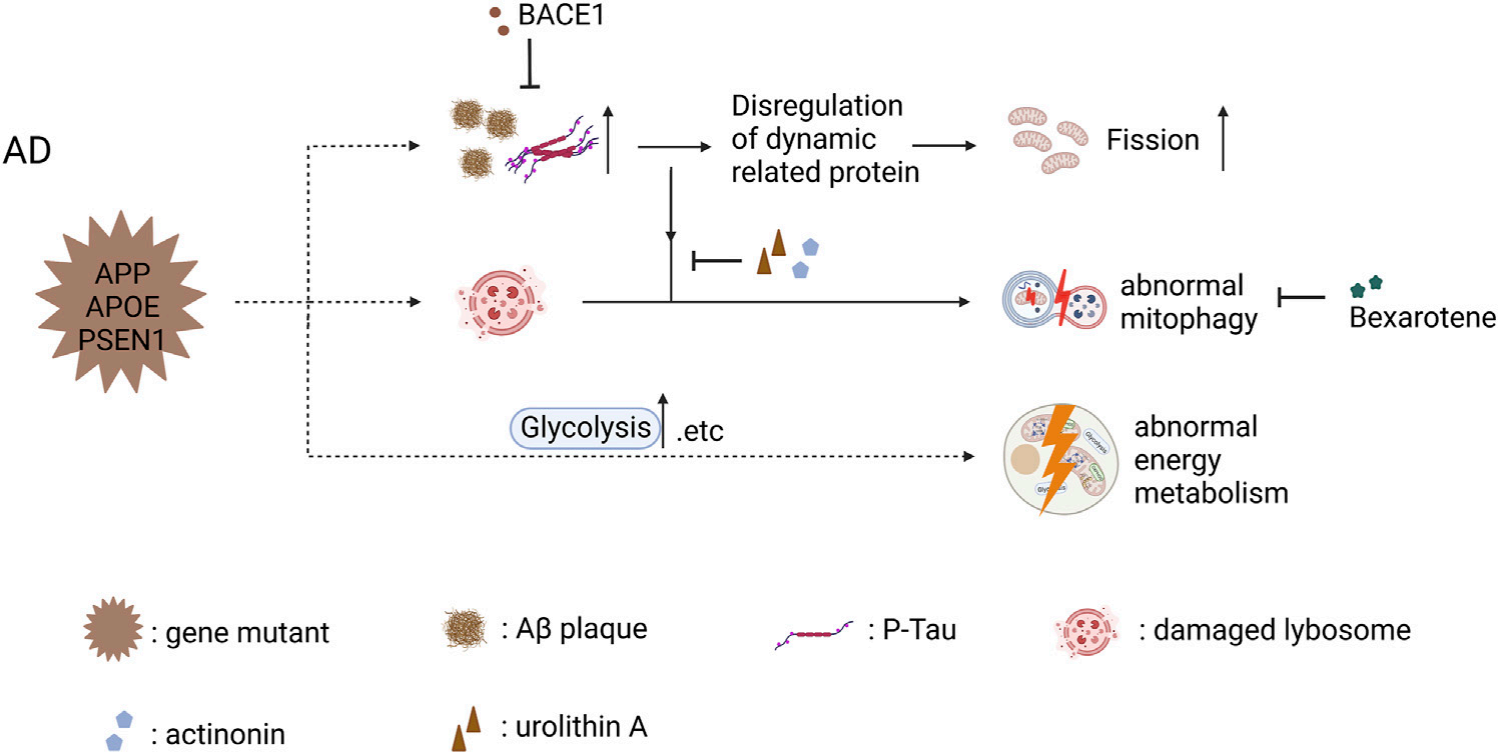
Mitochondrial dysfunction in AD iPSC-derived neurons (carrying APP, APOE, and PSEN1 mutant) and therapeutic strategies (created with BioRender.com). Abnormal accumulation of Aβ and p‐Tau disrupted the balance of mitochondrial fusion and fission and increased mitochondrial fission in cortical neuron carrying PSEN1 mutant, and BACE1 inhibited the abnormal accumulation of Aβ and p‐Tau to ameliorate mitochondrial fission. Abnormal accumulation of Aβ and p‐Tau decreased the levels of activated mitophagy proteins, leading to the abnormal mitophagy in AD iPSC-derived cortical neurons; actinonin, urolithin A and bexarotene are able to restore abnormal mitophagy through their own mechanism. AD iPSC-derived astrocytes showed abnormal energy metabolism including increased mitochondrial respiration and elevated glycolytic activity.

**FIGURE 3:**
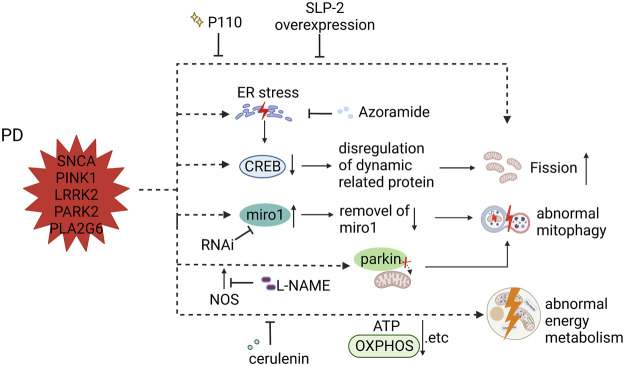
Mitochondrial dysfunction in PD iPSC-derived DA neurons (carrying SNCA, PINK1, LRRK2, PARK2, and PLA2G6 mutant) and therapeutic strategies (created with BioRender.com). DA neurons carrying PARK2 and LRRK2 mutants showed fragmented mitochondria, which was compensated by SLP-2 overexpression and P110. DA neurons with PLA2G6 mutation induced ER stress that facilitated the unfolded protein response (UPR), resulting in decreased signaling of CREB and final mitochondrial fragments; treatment with azoramide alleviated mitochondrial fission by enhancing the CERB signaling. PINK1 mutant iPSC-derived DA neurons prevented parkin from translocating to mitochondrial membrane, resulting in impaired mitophagy; L-NAME ameliorated damaged mitophagy by inhibiting the production of endogenous NOS. Accumulation of miro1 delayed the initiation of mitophagy in both LRRK2 and SCNA iPSC-derived DA neurons, and decreasing the level of miro1 protein by RNAi promotes mitophagy. DA neurons carrying PINK1 mutant displayed reduced levels of ATP production, which was improved by cerulenin through rescuing PINK1 deficiency.

**FIGURE 4:**
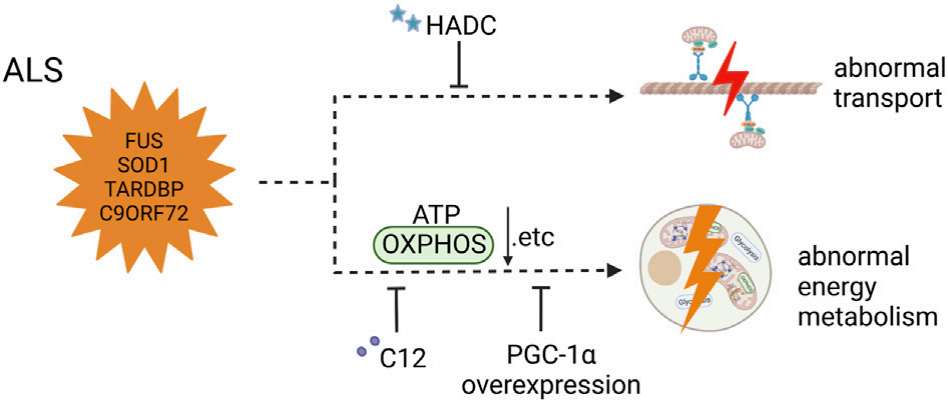
Mitochondrial dysfunction in ALS iPSC-derived neurons (carrying FUS, SOD1, and TARDBP mutant and C90ORF72 expanded GGGGCC repeats) and therapeutic strategies (created with BioRender.com). Mitochondrial transport defects were solved by HDAC inhibitors in MNs carrying FUS mutant. MNs with C9orf72 mutation showed impaired basal and maximal mitochondrial respiration, which was restored by overexpression of PGC1α. MNs carrying SOD1, TDP43, and C9ORF72 mutants and 3sALS mutants had reductions in basal respiration, ATP production, and spare respiratory, and treatment with C12 ameliorated the abnormal energy metabolism.

This review showed different mitochondrial dysfunction in AD, PD, and ALS, along with feasible treatments ameliorating these mitochondrial defects or even inhibiting disease progress (Figures 2–4). Mitochondrial dysfunction is one of the early pathological features of NDDs (Lin and Beal, 2006). Moreover, the impairment of mitochondria can directly threaten neural survival and trigger neurodegeneration, and the deteriorating microenvironment of neurodegeneration in turn exacerbates mitochondrial dysfunction, leading to the continued progression of NDDs (Onishi et al., 2021; Jetto et al., 2022). Therapeutic measures are divided into two types those that target pathways and those that target sources—for example, targeting a molecule in the disease signaling pathway or targeting compensation for damaged mitochondria to correct mitochondrial dysfunction. However, extensive animal experiments and preclinical trials are required to confirm the safety and efficacy of drugs before these therapeutic strategies can be translated into clinical drugs.

In conclusion, the use of iPSC-based modeling to characterize mitochondrial dysfunction in NDDs reviewed in this paper can provide a reference for other research studies, and these modeling methods provide an effective platform for mechanistic research and drug screening in NDDs. In the future, developing more effective and affordable treatment strategies may improve the quality of life for the majority of NDD patients.
